# Potassium Application Enhanced Plant Growth, Mineral Composition, Proximate and Phytochemical Content in *Trachyandra divaricata* Kunth (Sandkool)

**DOI:** 10.3390/plants11223183

**Published:** 2022-11-21

**Authors:** Bakholise Bulawa, Avela Sogoni, Muhali Olaide Jimoh, Charles Petrus Laubscher

**Affiliations:** Department of Horticultural Sciences, Faculty of Applied Sciences, Cape Peninsula University of Technology, Bellville, Cape Town 7535, South Africa

**Keywords:** Asphodelaceae, functional food, inflorescent vegetables, nutraceuticals, potassium sulphate, underutilized vegetables

## Abstract

Wild leafy vegetables are commonly included in the diet of people in rural homesteads. Among various wild edible vegetables in South Africa, *Trachyandra divaricata* (Sandkool) is one of the most abundant but underutilized due to the dearth of literature on its cultivation and nutritional value. In the present study, the effect of potassium application and pruning on growth dynamics, mineral composition, and proximate and phytochemical content in *T. divaricata* were evaluated. Treatments consisted of three potassium concentrations (0.0072, 0.0144, and 0.0216 M) supplemented in the form of potassium sulphate (K_2_SO_4_) with four pruning levels (unpruned, 5, 10, and 15 cm) applied in each treatment. The potassium doses were added to the nutrient solution, while the control treatment was sustained and irrigated with nutrient solution only. The results revealed a significant increase in flower bud yield, height, total dry and wet weight of shoots and roots, as well as ash and neutral detergent fibre in plants irrigated with 0.0072 M of K_2_SO_4_ without pruning. Conversely, chlorophyll content and Ca were comparable among treatments, while the highest yield of Na, P, N, and Zn was recorded in treatment 100 mL of K_2_SO_4_ with 10 cm pruning. Likewise, the highest antioxidant value (Polyphenols, Flavonol and DPPH) was obtained from plants irrigated with 0.0072 M of K_2_SO_4_ with 10 cm pruning. Based on these findings, *T. divaricata* is a promising leafy vegetable as a minimum dose (0.0072 M) of K with moderate pruning optimised its productivity in terms of growth, biomass parameters, nutritional content, and antioxidant potential. Due to its rich nutritional value, the plant should be domesticated and studied further for its potential nutraceutical benefits.

## 1. Introduction

Wild leafy vegetables are important food crops because they contain adequate amounts of nutrients, vitamins, and minerals for humans [1]. African communities have a long history of supplementing their diets with traditional vegetables [2,3]. In South Africa, most rural residents rely on foraged leaves as their primary source of leafy vegetables [4]. Although wild leafy vegetables are commonly consumed in rural areas, they are also consumed by urban residents who either buy them from traders or collect them in the wild [5,6]. 

Among various wild edible vegetables in South Africa, *Trachyandra divaricata* (Sandkool) is one of the most abundant but underutilized herbs due to the dearth of literature on its cultivation and nutritional value [7]. Although it was found in the early years in some parts of Australia, *T. divaricata* is native to South Africa. The plant is an invasive species within Asphodelaceae, and it has rhizomes which produce numerous fleshy leaves which spread along the surface up to 1 m long and a horizontal stem-like structure that produces white and purple flowers which turn into fruit sets at the tip, at the later stage [8]. Ethnobotanical reports claim that the inflorescence of this plant is palatable as it was consumed by the indigenous Khoisan people during times of famine [9,10]. To date, there are no studies that have been conducted on the cultivation, nutrition, and medicinal potential of this species, and the possible influence of potassium ions and pruning as growth enhancers when applied to improve the vegetative characteristics, nutritive properties, phytochemicals, and antioxidant potential of *T. divaricata* with a more focus on the inflorescence due to its documented edibility. 

Potassium (K) plays an important role in plant growth and development, making it a point of interest in maximizing crop yield [11]. This macro-nutrient has been reported to activate numerous important enzymes involved in protein synthesis, sugar transport, nitrogen metabolism, photosynthesis, as well as water balance and meristematic tissue growth in plants [12]. The availability of potassium fertilizers to crops affects not only production but also the quality of the harvested yield [13]. These qualitative characteristics are particularly important for leafy vegetables, both for industrial use and for household consumption. 

Moreover, flavour in numerous vegetables is determined by the number of solids, primarily sugars and organic acids, as well as volatile compounds whose biosynthesis and concentrations are influenced by potassium application [14]. Likewise, growing indications recommend that augmenting the K nutrition status of the plant can accord to abiotic stress tolerance by reducing the reactive oxygen species (ROS) level of the plants [15]. Concurrently, the application of potassium increased the efficiency of total chlorophyll in *Brassica juncea* cultivars under different irrigation regimes, resulting in an increase in photosynthetic efficiency, plant growth, total phenols, flavonoids, and vitamin C [16]. 

In addition to potassium supplementation, pruning is regarded as an essential operation in producing quality yield and early fruit in plants [17]. Recently, Ref. [18] reported that pruning of stems is an agronomic management practice associated with improved productivity of vegetables under protected conditions. This phenomenon was substantiated on cucumber (*Cucumis sativus* L.), where shoot pruning and potassium application resulted in increased production and yield [19]. Likewise, shoot pruning has been reported to increase the growth and production of tomatoes, watermelons, and sweet potatoes [20]. This then suggests that potassium and pruning are crucial for growth, quality of post-harvest yield and nutrition in plants and may be applied to improve the functional food status of a plant. 

The lack of literature on the cultivation, phytochemical and antioxidant activity and nutritional value of *T. divaricata* contributes to its underutilization and consumption among South African households. This study aims to investigate the effect of pruning and potassium dosages on vegetative growth, nutrient uptake, phytochemical content and antioxidants potential of flower buds of *T*. *divaricata* grown in hydroponics to find a suitable growth protocol for the plant under a hydroponic system. These findings are expected to serve as a model for future researchers, households, and potential farmers who may be interested in utilizing this plant for diet diversity and pharmaceutical precursors.

## 2. Results

### 2.1. Effect of Potassium and Pruning Levels on Plant Growth

#### 2.1.1. Height and Number of Flower Buds

The results obtained from this study indicated that various potassium concentrations, pruning levels and their interaction significantly (*p* ≤ 0.05) influenced the plant height and the number of flower buds (Table 1). The highest mean values for both height and flower buds’ number were recorded in the 0.0072 M treatment of potassium with no pruning, although an equivalent mean height was observed under the control treatment (0 M K_2_SO_4_). In contrast, the 0.0144 M and 0.0216 M of potassium with pruning levels recorded the smallest values in height and flower bud number (Table 1).

#### 2.1.2. Fresh and Dry Weight of Flower Buds

Both fresh and dry weights of flower buds significantly differed between treatments (Table 1). The highest fresh weight was obtained at 0.0072 M K_2_SO_4_ with unpruned and 15 cm pruning levels, respectively. These were significantly higher than most treatments, including the control (Table 1). The lowest fresh weight was obtained at 0.0216 M + 5 cm pruning level. The same trend was observed in the dry flower buds, where the highest mean weight was again obtained at 0.0072 M + 15 cm pruning level and 0.0072 M in unpruned treatments, respectively. The lowest dry weight of dry flower buds was obtained at a 0.0216 M dosage of K_2_SO_4_ pruned to 5 cm, 10 cm, and 15 cm. This was significantly low compared to the other treatments, including the control (Table 1). 

#### 2.1.3. Fresh and Dry Weight of Shoots

Experimental results also showed that potassium and pruning levels significantly (*p* ≤ 0.05) affected the fresh and dry weight of *T. divaricata* shoots (Table 2). The highest value of the fresh weight of shoots was obtained at 0.0072 M K_2_SO_4_ without pruning, and this was significantly higher than most treatments, including the control but did not differ significantly from 0 M with 15 cm pruning. The same trend was observed for the dry weight, where the heaviest weight was obtained at 0.0072 M K_2_SO_4_ without pruning, and this was significantly higher than most treatments, including the control but did not differ significantly from 0 M with 15 cm pruning (Table 2).

#### 2.1.4. Fresh and Dry Weight of Roots

Potassium application positively influenced the fresh and dry weights of *T. divaricata* roots. The highest fresh weight of roots was obtained at 0.0072 M K_2_SO_4_ with no pruning. This was significantly higher than most treatments, including the control but did not differ significantly from treatment 0.0144 M K_2_SO_4_ with 5 cm pruning. The heaviest dry weight of the roots was again recorded at 0.0072 M K_2_SO_4_ with no pruning. However, this was not significantly higher than the control, 0 M K_2_SO_4_ + 15 cm and 0.0072 M K_2_SO_4_ + 15 cm pruning, respectively. 

### 2.2. Effect of Potassium Dosage and Pruning Levels on Chlorophyll Content

**Figure 1:**
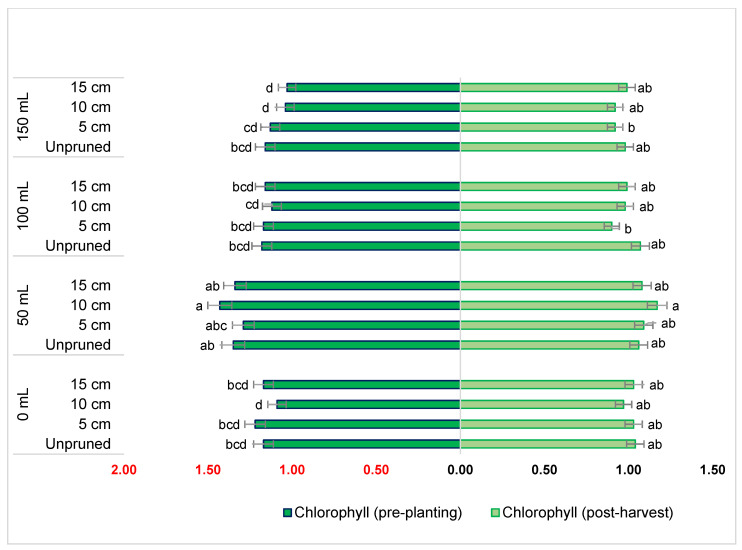
The effect of potassium and pruning levels on chlorophyll content of hydroponically grown *T. divaricata*. Note: Means that do not share a letter are significantly different at α = 0.05. The potassium dosages of 0 M, 0.0072 M, 0.0144 M and 0.0216 M are respectively equivalent to 0 mL, 50 mL, 100 mL, and 150 mL.

The results obtained from this experiment suggest that varying potassium concentration and pruning (only at pre-planting) had a significant effect on the chlorophyll content of *T. divaricata*, while pruning levels and their interaction with potassium application had no significant effect (Figure 1). The highest mean value of chlorophyll content at pre-planting was recorded in a combination of 0.0072 M K_2_SO_4_ with 10 cm pruning. This was significantly different from other treatments except for 0.0072 M K_2_SO_4_ at all pruning levels. However, at post-harvest, the highest mean value was obtained at 0.0072 M with 10 cm pruning, and this was statistically similar to many treatments, including the control but differed significantly from treatment 0.0144 M + 5 cm and 0.0216 M + 5 and 10 cm pruning levels (Figure 1).

### 2.3. Effect of Potassium and Pruning on Mineral Composition of Flower Buds of T. divaricata

#### 2.3.1. Macronutrients

Table 3a shows macronutrients in flower buds of hydroponically grown *T. divaricata* under varying potassium concentrations and pruning levels. The major elements tested were calcium (Ca), magnesium (Mg), sodium (Na), phosphorus (P), nitrogen (N) and potassium K). The increasing order of mineral yields per 100 g of plant sample is Na < P < Mg < Ca < K.

Potassium application, pruning levels, and their interaction did not have any significant effect on the Ca yield of the flower buds in all tested treatments, including the control, while Mg was significantly affected by the treatments (Table 3a). The highest Mg yield was recorded in harvests from treatment 0 M (K_2_SO_4_) +10 cm pruning, and this was statistically similar to the control, 0 M + 5 and 15 cm pruning levels, respectively. On the contrary, the Na composition of the samples varied in the treatments, and the highest yield was recorded in treatment 0.0144 M + 10 cm pruning, which was significantly higher than all treatments, including the control. Likewise, the highest P composition was recorded in treatment 0.0144 M + 10 cm pruning. However, this was not significantly different from the control, 0 M + 5 cm, 10 cm and 0.0072 M + 5 cm, 10cm and 15 cm pruning, respectively. Moreover, the highest N yield was recorded in treatment 0.0144 M with the following pruning levels (unpruned, 5 cm, and 10 cm pruning). These values were significantly higher than all other treatments, including the control. Conversely, the highest K yield was recorded in treatment 0.0072 M with no pruning, and this was significantly higher than all treatments, including the control (Table 3a). 

#### 2.3.2. Micronutrients

The level of accumulation of heavy metals such as zinc (Zn), manganese (Mn), copper (Cu) and iron (Fe) in the evaluated flower bud samples are presented in Table 3b. This study showed a significant increase in the accumulation of Zn and Mn in response to potassium and pruning levels in *T. divaricata* flower buds, although the Zn content was not significantly changed by K-treatments, especially in unpruned samples. The highest yield of Zn was recorded in treatment 0.0144 M with 10 cm pruning, and this was significantly higher than all treatments, including the control. Likewise, the highest Mn yield obtained in treatment 0.072 M with no pruning was significantly higher than all other treatments, including the control. However, this was not the case for Cu, where the highest yield (0.53 mg/100 g) obtained from treatment 0.0216 M with no pruning did not differ significantly from the control, 0 M with 15 cm and 0.0216 M with 15 cm pruning, respectively. The same trend was observed for Fe, where the highest yield (13.3 mg/100 g) recorded from treatment 0.0144 M with 15 cm pruning did not differ statistically from the control and 0.0144 M with no pruning treatment (Table 3b).

### 2.4. Effect of Potassium and Pruning on Proximate Composition of Flower Buds

This study revealed significant differences in proximate contents in flower buds of hydroponically grown *T. divaricata* under varying potassium concentrations and pruning levels regarding moisture, neutral detergent fibre, crude fat, ash, crude protein, non-fibre-carbohydrate, and energy values (Table 4). The highest ash content of the flower buds was recorded in treatment 0.0072 M without pruning and 0.0072 M with 15 cm pruning, respectively. These values were significantly higher than all other treatments, including the control. On the contrary, the highest crude fat content was obtained from treatment 0.0144 M with 15 cm pruning. However, this did not differ from the control, 0 M + 5 cm, 0.0072 M + 10 cm, 0.0144 M with no pruning and 0.0216 M with no pruning, respectively. Moreover, the highest yield of crude protein was recorded in treatment 0.0144 M with 5 cm pruning, and this was significantly higher than all treatments, including the control. Similarly, the highest NDF (37.8%) was significantly higher than all treatments, including the control (Table 4).

The NFC percentage was high in the 0.0216 M treatment with 5 cm pruning, and this was comparable with the 0.0216 M treatment with all pruning levels. When looking at the moisture percentages, the highest yield was recorded in the 0.0074 M treatment with 10 cm pruning. This was not significantly different from the 0.0072 M treatment with 15 cm pruning and 0.0144 M with 5 cm pruning, respectively (Table 4). The energy value of flower buds of *T. divaricata* yields estimated in (KJ/100 g) varied among treatments. Analysis of results obtained shows that plants pruned at 15 cm without the application of potassium (0 M) recorded the highest energy density but were comparable to many treatments, including the control. 

### 2.5. Effect of Potassium and Pruning Levels on Phytochemicals and Antioxidant Activity of Flower Buds

#### 2.5.1. Total Polyphenols

Potassium supplementation, pruning and their interaction had a significant effect on the accumulation of polyphenols in the flower bud of *T. divaricata* (Table 5). The combination of 0.0144 M without pruning recorded the highest concentrations of polyphenols (6.57 mg GAE/g), while 0.0144 M + 5 cm had the least value (2.69 mg GAE/g). The highest mean value was significantly higher than the control and other treatments but did not differ from treatment 0.0072 M with all pruning levels.

#### 2.5.2. Total Flavonol

Different potassium concentrations, pruning levels and their interaction have shown a significant (*p* ≤ 0.05) effect on the flavonol content of *T. divaricata* flower buds. The highest mean value of flavonol was recorded in the 0.0072 M treatments with 10 cm pruning (Table 5). However, this did not differ from the 0.0144 M treatment with 15 cm pruning and 0.0072 M with 15 cm pruning, respectively. Treatment “0 M” with 5 cm pruning produced the lowest flavonol values when compared to other treatments, including the control.

#### 2.5.3. FRAP Antioxidant Content

The current results revealed the significant effect of different potassium concentrations and pruning levels on the FRAP activity of the flower buds of *T. divaricata*. The 0.0072 M treatment with 5 cm pruning had the highest FRAP activity (33.69 µmol AAE/g) when compared to other treatments, including the control. This was significantly higher than all treatments, while the 0.0144 M treatment without pruning had the least value (10.09 µmol AAE/g; Table 5).

#### 2.5.4. DPPH Antioxidant Content

The effect of potassium, pruning levels as well as their interaction had a significant effect on the DPPH activity of the flower buds of *T. divaricata.* The 0.0072 M treatment with 10 cm pruning recorded the highest DPPH antioxidant (11.47 µmol TE/g). This was significantly higher than all other treatments, including the control. The least DPPH antioxidant (3.54 µmol TE/g) was recorded under treatment “0.0144 M” without pruning.

#### 2.5.5. TEAC/ABTS Activity

A different trend was observed in the TEAC/ABTS activity, where the 0 M treatment with 15 cm pruning recorded the highest activity (19.04 µmol TE/g). This was significantly higher than all treatments, including the control. The least ABTS activity (2.69 µmol TE/g) was recorded under the 0 M treatment with 5 cm pruning.

## 3. Materials and Methods

### 3.1. Experimental Location

The experiment was carried out at the research greenhouse of the Cape Peninsula University of Technology (CPUT), Bellville campus, Cape Town, South Africa, located at 33°55′56′′ S and 18°38′25′′ E. The greenhouse was equipped with environmental control with a temperature range of 21–26 °C during the day and 12–18 °C at night, with relative humidity averages of 60%. The average daily photosynthetic photon flux density (PPFD) was 420 μmol/m^2^/s, and the maximum was 1020 μmol/m^2^/s.

### 3.2. Plant Preparation and Experimental Design

Plant material of *T. divaricata* was obtained from the CPUT nursery. The plant material was propagated by the division technique as described by Ngxabi et al. [7]. Divided plantlets were transplanted into 12.5 cm filled with inert, silica sand grade 6/17 media which were then harden-off for a week under a shade cloth. Thereafter the plants were pruned at varying heights (5, 10 and 15 cm) before they were moved to the experimental site.

**Figure 2:**
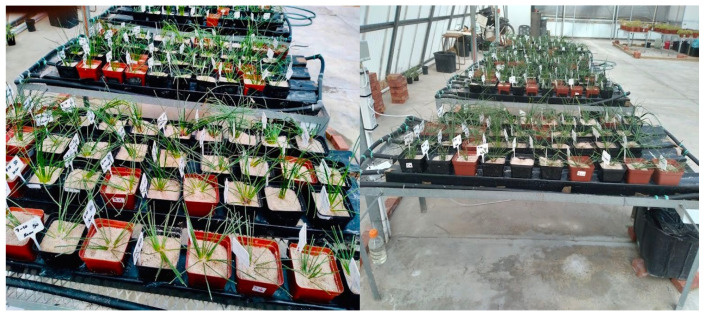
The experimental layout showing replicates (*n* = 10) in the Nutrient Filter Technique hydroponic system (Picture: B. Bulawa).

The hydroponic experiment was conducted as described by Ngxabi et al. [7] and [21] with slight modifications. Four identically constructed nutrient film technique (NFT) systems were used, with each system on separate wire mesh square tables (2.5 m) that provided a flat surface (Figure 2). The NFT systems were labelled as T1–T4. Each system had its low-density polyethene (LDPE) 50 L reservoir in which the nutrient solution was prepared. There were 4 Polyvinyl Chloride (PVC) square gutters (2 m) put in place with cable ties on each table, in which 4 different pruning heights and fertigation treatments were tested (Table 6). The gutters were sealed with PVC adhesive to prevent leaks. In the construction of each system, a 1 × 2000 L/h submersible pump with 2.5 m head capacity, 20 mm LDPE irrigation piping, 4 × 20 mm elbow irrigation fittings and 4 × 20 mm flow regulators were used (Figure 2).

### 3.3. Treatments

Nutrifeed fertiliser (10 g/5 L) obtained from Starke Ayres, Cape Town, was used as the standard fertilizer and as a source of base nutrients for all treatments since it is mostly used by South African vegetable growers. The Nutrifeed fertiliser contained the following ingredients: N (65 mg/kg), P (27 mg/kg), K (130 mg/kg), Ca (70 mg/kg), Cu (20 mg/kg), Fe (1500 mg/kg), Mo (10 mg/kg), Mg (22 mg/kg), Mn (240 mg/kg), S (75 mg/kg), B (240 mg/kg), and Zn (240 mg/kg). Potassium was supplemented in the form of potassium sulphate and injected into the nutrient solution at varying ratios in different treatments (0.0072, 0.0144 and 0.0216 M). The control was sustained and irrigated with Nutrifeed only.

### 3.4. Determination of Plant Growth

#### 3.4.1. Shoot Length and the Number of Flower Buds

The shoot length and the number of flower buds were used as indicators of new growth. Shoot length was measured every two weeks with a metal tape measure from the substrate level to the tip of the tallest shoot, while the number of flower buds was counted manually. 

#### 3.4.2. Plant Weight

To establish homogeneity within the samples used for cultivation, the weight of the plants was determined using a standard laboratory scale (RADWAG^®^Model PS 750.R2). Post-harvest, shoots, flower buds, and roots were separated, and the fresh/wet weights of the individual samples were recorded. The plant material was then oven-dried at 55 °C in a LABTECH™model LDO 150F (Daihan Labtech India. Pty. Ltd., 3269 Ranjit Nagar, New Delhi, India) to a constant weight and recorded. The difference between the fresh and dry weight was compared with the amount of water held within the plant tissues. 

### 3.5. Chlorophyll Content

The chlorophyll content was measured using a Soil Plant Analysis Development (SPAD-502) meter supplied by Konica Minolta, Cape Town, South Africa. The readings of two fully formed leaves were taken from each plant, and the figures were averaged out by the SPAD-502 meter to produce a final number [22].

### 3.6. Mineral Analysis

The mineral composition of each set of replicates in the experiment was determined using the Inductively Coupled Plasma-Optical Emission Spectrometer in the analytical laboratory of the Department of Agriculture and Rural Development, KwaZulu Natal Province was used to carry out the elemental analysis as described by [23].

### 3.7. Proximate Analysis

#### 3.7.1. Moisture Content

The moisture content was determined using slightly modified procedures described by [22]. Empty porcelain vessels were oven-dried at 105 °C for one hour, cooled in a desiccator, and weighed W1. About 1 g (W2) of the pulverised samples of *T. divaricata* was placed in a vessel and oven-dried to a constant weight at 105 °C. The vessel and its contents were cooled in a desiccator before being re-weighed (W3). The percentage of moisture content was calculated using the equation below.

$$\%\;\mathrm{Moisture}\;\mathrm{content}=\frac{W2-W3}{W2-W1}\times 100$$

#### 3.7.2. Crude Fibre Content

This was evaluated as described by [24,25] with slight modifications. About 2 g of pulverised samples was boiled in 100 mL of 1.25% concentrated H_2_SO_4_ for 30 min in a digestion tube, then filtered under pressure. The digested residue was washed several times with boiling water until a clear mixture was obtained, then rinsed with 100 mL of 1.25% NaOH solution. The resulting residue was then dried at 100 °C, cooled in a desiccator, and weighed (F1). Following that, the residues were incinerated for 5 h in a muffle furnace at 550 °C, cooled in a desiccator, and re-weighed (F2). The crude fibre percentage was estimated as

$$\%\;\mathrm{Crude}\;\mathrm{fibre}=\;\frac{F1-F2}{\mathrm{Original}\;\mathrm{weight}\;\mathrm{of}\;\mathrm{the}\;\mathrm{pulverised}\;\mathrm{sample}}\times 100$$

#### 3.7.3. Crude Fat Content

The crude fat was calculated in accordance with the Association of Official Analytical Chemists [24] guidelines. About 1 g of pulverized sample was extracted in 100 mL of diethyl ether and shaken on an orbital shaker for 24 h. The mixture was then filtered, and the filtrate was collected in previously weighed clean beakers. The ether extract was then equilibrated with 100 mL diethyl ether, shaken for another 24 h on an orbital shaker, and the filtrate was collected in a beaker (W1). The ether filtrate was concentrated to dryness in a steam bath and oven-dried at 55 °C before being reweighed in the beaker (W2). As a result, the crude fat content was calculated as

$$\%\;\mathrm{Crude}\;\mathrm{fat}\;\mathrm{content}=\frac{W2-W1}{\mathrm{original}\;\mathrm{weight}\;\mathrm{of}\;\mathrm{the}\;\mathrm{pulverised}\;\mathrm{sample}}\;\times 100$$

#### 3.7.4. Ash Content

The protocol developed by [24] was used to determine the percentage ash content of plant samples. Porcelain crucibles were oven-dried at 105 °C for 1 h after being labelled with sample codes using a heat-resistant marker. The crucibles were weighed after cooling in a desiccator (W1). Thereafter, 1 g of ground samples were placed in pre-weighed porcelain crucibles and reweighed (W2). The crucibles with the contents were placed in a muffle furnace set to 250 °C for 1 h and then 550 °C for 5 h to completely ash the samples. After cooling in a desiccator, the samples were weighed (W3). The ash content of the samples was calculated as

$$\%\;\mathrm{Ash}\;\mathrm{content}=\frac{W2-W3}{W2-W1}\times 100$$

#### 3.7.5. Crude Protein

This was determined by boiling 2 g of ground samples at 420 °C in a Kjeldahl flask for 45 min with concentrated H_2_SO_4_ (20 mL) until a clear mixture was obtained, with Kjeldahl digestion tablet (0.15 g of copper sulfate, 5.0 g of potassium sulfate and 0.15 g titanium oxide) acting as a catalyst [25,26]. The digested extracts were distilled after being filtered and dissolved in 250 mL. The aliquot containing 50 mL of 45% NaOH was distilled further in a 500 mL round-bottomed flask, and 150 mL of the distillate was transferred into a flask containing 100 mL of 0.1 M HCl. This was then titrated with methyl orange against 2.0 mol/L NaOH. The endpoint of titration was indicated by a yellow colour change, and the percentage nitrogen content was calculated as shown in the equation below.

$$\mathrm{Crude}\;\mathrm{Protein}=\;\frac{\left[\left(\mathrm{ml}\;\mathrm{std}\;\mathrm{acid}\;\times \;N\;\mathrm{of}\;\mathrm{acid}\right)-\left(\mathrm{ml}\;\mathrm{bank}\;\times \;N\;\mathrm{of}\;\mathrm{base}\right)\right]-\left(\mathrm{ml}\;\mathrm{std}\;\mathrm{base}\;\times \;N\;\mathrm{of}\;\mathrm{base}\right)\times 1.4007}{\mathrm{original}\;\mathrm{weight}\;\mathrm{of}\;\mathrm{the}\;\mathrm{pulverised}\;\mathrm{sample}}$$

where N = normality and the percentage of crude protein were obtained by multiplying the nitrogen value by a constant factor of 6.25 [27].

#### 3.7.6. Neutral Detergent Fibre (NDF)

The NDF composition of the samples was determined using the equation below, as described by (Idris et al., 2019).

$$\%\;\mathrm{NDF}=\frac{\left(W1+W2\right)-W1}{\mathrm{Weight}\;\mathrm{of}\;\mathrm{the}\;\mathrm{sample}}\times 100$$

#### 3.7.7. Non-Fibre Carbohydrate (NFC)

The below formula was used to calculate the sample’s non-fibre carbohydrate content.

$$\%\;\mathrm{NFC}=100-\left(\%\;\mathrm{Ash}+\%\;\mathrm{Crude}\;\mathrm{fat}+\%\;\mathrm{Crude}\;\mathrm{protein}+\%\;\mathrm{NDF}\right)$$

#### 3.7.8. Energy Content

The energy content of each sample of *T. divaricata* from different treatments was estimated by adding the multiplied values for total carbohydrate, crude lipid (excluding fibre), and crude protein using factors (17 KJ, 37KJ, and 17 KJ) using the conversion factor proposed by FAO [28].

$$\mathrm{Energy}\;\mathrm{content}\;(\mathrm{KJ}/100\;\mathrm{mg})=\left(\mathrm{CHO}\times 17\right)+\left(\mathrm{Crude}\;\mathrm{fat}\times 37\right)+\left(\mathrm{Crude}\;\mathrm{protein}\times 17\right)$$

where CHO denotes total carbohydrate as determined by the equation below [29]:

CHO = NFC + NDF

### 3.8. Phytochemicals and Antioxidant Assays

#### 3.8.1. Sample Preparation

Harvested flower bud materials were immediately dried in a fan-drying laboratory oven at 40 °C for 7 days. The dried material was ground into a fine powder using a Junkel and Kunkel model A 10 mill. It was then extracted by mixing 100mg of the dried powdered material with 25 mL of 80% (*v/v*) ethanol (EtOH; Merck, Modderfontein, South Africa) for 1 h. It was centrifuged at 4000 rpm for 5 min, and the supernatants were used for all analyses.

#### 3.8.2. Polyphenol Assay

Total polyphenols assay (Folin & Ciocalteu’s assay) was performed as described by [30]. Folin & Ciocalteu’s phenol reagent (2N, Sigma South Africa, Sandton, South Africa) was diluted 10 times with distilled water, and a 7.5% sodium carbonate (Sigma, South Africa) solution was prepared. In a 96-well plate, 25 μL of the crude extract was mixed with 125 μL Folin & Ciocalteu’s phenol reagent and 100 μL sodium carbonate. The plate was incubated for 2 h at room temperature. The absorbance was then measured at 765 nm in a Multiskan spectrum plate reader (Thermo Electron Corporation, Waltham, MA, USA). The samples’ polyphenol values were calculated using a gallic acid (Sigma, South Africa) standard curve with concentrations varying between 0 and 500 mg/L. The results were expressed as mg gallic acid equivalents (GAE) per g dry weight (mg GAE/g DW).

#### 3.8.3. Estimation of Flavonol Content

The flavonol content of the extracts was determined using quercetin 0, 5, 10, 20, 40, and 80 mg/L in 95% ethanol (Sigma-Aldrich, Johannesburg, South Africa) as standard. For each sample, about 12.5 μL of crude sample extracts were mixed with 12.5 μL 0.1% HCl (Merck, South Africa) in 95% ethanol and 225 μL 2% HCl [31,32]. The extracts were then incubated at room temperature for 30 min. The absorbance was read at 360 nm at a temperature of 25 °C. The results were expressed as mg quercetin equivalent per g dry weight (mg QE/g DW).

#### 3.8.4. Ferric Reducing Antioxidant Power (FRAP) Assay

The FRAP assay was performed using the method of [33] as described by [34]. FRAP reagent was prepared by mixing 30 mL Acetate buffer (0.3M, pH 3.6) (Merck, South Africa) with 3 mL 2,4,6- tripyridyl-s-triazine (10mM in 0.1M Hydrochloric acid), 3 mL Iron (III) chloride hexahydrate (FeCl_3_·6H_2_O) (Sigma, South Africa) and 6 mL of distilled water. In a 96-well microplate, 10 μL of the crude sample extract was mixed with 300 μL of the FRAP reagent and incubated for 30 min at room temperature. The absorbance was then measured at 593 nm in a Multiskan spectrum plate reader (Thermo Electron Corporation, Waltham, MA, USA). The FRAP values of the tested plant samples were calculated using an L-Ascorbic acid (Sigma-Aldrich, South Africa) standard curve with concentrations varying between 0 and 1000 μM. The results were expressed as μM Ascorbic acid equivalents (AAE) per gram dry weight (μM AAE/g DW) [35].

#### 3.8.5. DPPH Free Radical Scavenging Activity

A 0.135 mM DPPH solution prepared in a dark bottle was used to generate the DPPH radicals [36]. About 300 μL of DPPH solution was reacted with graded concentrations (0 and 500 μM) of Trolox standard (6-Hydrox-2,5,7,8-tetramethylchroman-2-20 carboxylic acid) solution and 25 μL of crude extract. After incubating the mixtures for 30 min, the absorbance was measured at 517 nm. The results were expressed as μM/Troloxequivalent per g dry weight (μM TE/g DW).

#### 3.8.6. ABTS Free Radical Scavenging Activity

The ABTS assay was performed following the method described by Jimoh et al. [30]. The stock solutions included a 7 mM ABTS and 140 mM potassium-peroxodisulphate (K_2_S_2_O_8_) (Merck, Modderfontein, South Africa) solution. The working solution was then prepared by adding 88 μL of K_2_S_2_O_8_ to 5 mL of ABTS solution. The two solutions were mixed well and allowed to react for 24 h at room temperature in the dark. Trolox (6-Hydrox-2,5,7,8-tetramethylchroman-2-20 carboxylic acid) was used as the standard, with concentrations ranging between 0 and 500 μM. Crude sample extracts (25 μL) were allowed to react with 300 μL of ABTS in the dark at room temperature for 30 min before the absorbance was read at 734 nm at 25 °C in a plate reader. The results were expressed as μM/Trolox equivalent per g dry weight (μM TE/g DW).

### 3.9. Statistical Analysis

The experimental data were analysed using two-way analyses of variance (ANOVA). Tukey’s least significant difference was used to compare means at *p* ≤0.05 level of significance between treatments. At α level of 0.05, means that do not share a letter are significantly different. All calculations were done on the computer software program STATISTICA version 13.5.0.17.

## 4. Discussion

Potassium (K) plays an important role in plant growth and development, making it a point of interest in maximizing crop yield [11]. It activates important enzymes that drive various physiological and metabolic mechanisms, including those involved in protein synthesis, sugar transport, nitrogen metabolism, and photosynthesis [37,38]. In the present study, plants supplemented with 0.0072 M of potassium without pruning had an improved plant height, number of flower buds as well as total yield. This is consistent with [20], who found that pruning resulted in shorter average stem growth than without pruning, and vice versa. These results are supported by previous studies that augmenting the K^+^ nutrition status of the plant can significantly accord to stress tolerance and improved growth and flowering [15]. The increase in height, the number of flower buds and plant biomass may be due to cell enlargement and a corresponding increase in nodal length caused by the increased availability of nutrients and growth substances due to higher photosynthate accumulation in response to potassium application. Likewise, Wanshnong et al. [39] reported the same findings on *Papaya* var. Red Lady, where the application of K^+^ increased the plant height, plant girth and the total number of leaves.

Furthermore, the additional application of K^+^ has been reported to enhance the photosynthetic CO_2_ fixation as well as the transportation and consumption of photoassimilates in plants which regulate chlorophyll content during oxidative stress conditions [40]. This was observed in this study, where pruning stress was mitigated by the application of K and did not have any significant effect on the chlorophyll content of *T. divaricata* leaves. Recently, Siddiqui et al. [41] also reported the same findings on tomato seedlings exposed to drought stress which was mediated by the exogenous application of K that further regulated endogenous K^+^, which enhanced the chlorophyll content and photosynthesis in tomato seedlings. Concurrently, Rani et al. [16] also alluded to this phenomenon in *Brassica juncea* cultivars under different irrigation regimes, where the application of potassium increased the efficiency of total chlorophyll resulting in an increase in photosynthesis. 

The application of fertilizers has been reported to cause changes in the uptake and concentrations of other nutrients available to plants [42]. This may be due to interactions in ion uptake and or transport of other minerals within the plant cells [43,44]. Hence, it is very crucial to apply the correct doses of fertilizers for plant growth and development. Plant-based minerals are an important component of the human diet. They sustain life by supplying vital nutrients required for the body’s psychophysical well-being [45]. The highest mineral contents found in this study support previous reports that wild vegetable species are a source of essential minerals [46]. Variations in the results obtained in this study further showed that there was an antagonistic effect between K, Calcium (Ca), and Magnesium (Mg) ions since the additional application of K caused a decrease in Ca and Mg. Although these minerals were slightly decreased in 0.0072 M of potassium-treated samples with different pruning levels, they enhanced the growth and development of flower buds. These results substantiate earlier findings of Norozi et al. [44], where the application of potassium fertilizer decreased leaf concentrations of Calcium and Magnesium while Nitrogen, Phosphorus, Potassium and Sodium increased. These results have shown that the calcium content of *T. divaricata* fell short of the recommended daily allowance (RDA) of 1000 mg proposed for an adult. While magnesium content was well above the RDA value of 55 mg/100 g, as stated by USDA [27] in all tested treatments. Magnesium performs critical functions ranging from structural roles in nucleic acids, proteins, and polyribosomes to neurotransmitter release, cell adhesion, calcium-potassium homeostasis stabilization, and as an enzymatic cofactor [47]. Regular consumption of this plant will thus ensure optimal magnesium concentration in the serum.

The application of potassium did not, however, enhance the uptake of K^+^ in the treated samples, as the highest potassium content was recorded in the samples of *T. divaricata* treated with a moderate 0.0072 M of potassium. Likewise, potassium is an essential nutrient in a healthy diet. It is extremely important physiologically because it activates intracellular and extracellular cations required for the maintenance of blood pressure, muscular contractility, and nerve impulse conduction [47]. Earlier, Jimoh et al. [22] stated that the consumption of at least 2000 mg is desirable for an adult, which is abundant in *Trachyandra divaricata* flower buds in all tested variables, including the control. Sodium is the most abundant cation in extracellular fluid. It is essential for maintaining acid-base balance and acts as a precursor for nerve impulse transmission [48]. The RDA for sodium is between 320–500 mg as stipulated by USDA [27], of which none of the treatments met this requirement, whereas the RDA for phosphorus (700 mg/day) was met in samples of *T. divaricata* treated with 0.0072 M of potassium in all pruning levels. Phosphorus is an important nutrient in our diet and serves crucial functions as an important physiologic buffer, as a substrate for critical cellular functions, and along with calcium as a component of the bone mineral in the skeleton [49]. Hence the consumption of this vegetable will help strengthen the skeletal system of humans. 

Micronutrients such as iron, zinc, aluminum, copper, and manganese are variously important for human nutrition [5]. These trace elements are required in less than 20 mg per day and account for less than 0.01% of body weight [50]. The trace elements evaluated in flower buds of *T. divaricata* meet the recommended daily allowance except for copper, as reported by USDA [27]. The Iron (Fe) content of the treatments ranged from 5.6 to 13.3 mg/100 g, and this was lower than the values reported on other wild consumed vegetables in South Africa such as *Lecaniodiscus cupaniodes* (27.8 mg/100 g), *Sterculia tragacantha* (803.7 mg/100 g) and *Ipomea plebeian* (1225 mg/100 g) respectively [5]. Likewise, manganese recorded in the flower buds of *T. divaricata* ranged from 2.8 to 6.7 mg/100 g, and this was lower than the value reported in Moring oleifera (252 mg/100 g) [51]. Manganese functions as an antioxidant and is also involved in cellular reproduction, immune system function, blood sugar regulation, digestion, and bone growth [52]. While Fe is required for haemoglobin formation, lowering anaemia rates, and is also required for proper central nervous system function [53]. Based on these findings, it can be assumed that the flower buds of *T. divaricata* are safe for consumption since they accumulate a lower quantity of heavy metals as compared to other wild vegetables. 

The proximate composition of the flower buds of *T. divaricata* grown under varying potassium concentrations and pruning levels differed significantly in the present study. The ash content of food is a measure of its nutritional value and is thought to be a reflection of the mineral contents preserved in food materials [54]. The ash content of the analyzed flower buds was high compared to other reports on wild vegetables, which usually does not exceed 5%. The ash content of the flower bud ranged from 13.3% to 23.6% in all treatments, and this corresponds to the composition found in processed foods [23]. These findings are in tandem with that of Ntuli [5] on two water spinach species (*Ipomea plebeian* R.Br. and *Ipomea wightii* (Wall.) Choisy), where the ash content reported was 24%. This high ash value indicates that the plant is high in dietary fibres, which provide shelter for digestive organisms in the gastrointestinal tract [55]. Generally, wild vegetables such as *Amaranthus caudatus* L., *Solanum nigrum, lbertisia delagoensis, Ipomoea plebeia, Ipomoea wightii, Limeum sulcatum, and Pyrenacantha kaurabassana* have been found to have low levels of unsaturated fat of between 1.9–4.8% [5,22,50]. The fat content in flower buds of *T. divaricata* ranged from 1.7–2.3%, and this was similar to the findings of Ntuli [5]. The fat in leafy vegetables provides energy, essential fatty acids, and vitamins and which adds to palatability through absorption and retaining flavour. Protein values ranged from 21.7 to 26.6% in tested samples, and these were lower than those reported in *A. caudatus* (30.2%) and *S. nigrum* (38.98%), respectively [22,50]. Nevertheless, the protein content of the flower buds of *T. divaricata* was comparable with that of *Moringa oleifera* leaves (27.3%), as reported by Sultana [56].

The non-fibre carbohydrate (NFC) content ranged between 19.85–60.5% in all treatments, including the control. The highest carbohydrate content was found in treatment 0.0144 M with 5 cm pruning, and these values are higher than those reported by Bvenura et al. [57] on edible wild vegetables of the southern African region. This suggests that *T. divaricata* is a good source of carbohydrates and has a high caloric value that can help meet the body’s caloric needs. Carbohydrates are an essential component of a healthy diet, accounting for 50% of our daily calorie intake [56]. The moisture content of the flower buds ranged from 9.9–12.1% in tested treatments, including the control. These lower values suggest that the flower buds of this plant might have a long storage lifespan which will favour the growers and sellers, and its potential use for the improvement of the shelf life of foods or as preservatives may be investigated. 

Fruits and vegetables with high antioxidant capacity provide added-value products and are widely accepted by consumers and the food industry [58]. The consumption of plant products with high phenolic content can protect human tissue oxidation by scavenging free radicals and inhibiting lipid peroxidation, advancing the nutritional quality of food and avoiding potential problems caused by excessive consumption of synthetic additives [59]. Therefore, it is imperative to improve the phytochemical components in fruits and vegetables during cultivation. This can be achieved through the manipulation of growth conditions and fertilization. In the present study, the application of potassium significantly increased the phytochemical content of the flower buds, with more prominent content in plants irrigated with the lowest K concentration (0.0072 M), while the highest dose (0.0216 M K concentration resulted in the lowest antioxidative capacity and content of total phenols and flavonol. These results validate the findings of Mahmoud et al. [60] on *Solanum tuberosum* tubers, where the application of a moderate concentration of potassium increased the accumulation of phytochemicals. A similar trend was also reported by Zikalala et al. [61] on the nutritional quality of baby spinach (*Spinacia oleracea* L.) as affected by nitrogen, phosphorus, and potassium fertilisation, where the moderate application of K increased the concentrations of total phenols, total antioxidants activity, total flavonoids, and vitamin C. Therefore, these results have suggested that potassium application in vegetable crops may affect not only plant yield but also the synthesis of other biologically active substances, such as phenolics, which have a protective role against induced stress responses [62].

## 5. Conclusions

The current study established that potassium application and pruning levels had a positive impact on the growth, mineral composition, proximate contents, and antioxidant capacity of *Trachyandra divaricata*. Samples of *T. divaricata* supplemented with 0.0072 M of additional potassium with or without pruning accumulated elevated phytochemical, antioxidant, and nutritional compositions that are within the recommended daily allowance for consumption. The accumulation of high mineral nutrients in flower buds reflects a nutrient-supplying vegetable characteristic. The plant’s high fibre content confirms its digestive effectiveness in humans, and its high protein content confirms its worth as an immune booster, an important nutraceutical, and a potential functional food. Moreover, the lower moisture content suggests that the plant might have a long storage lifespan. Based on these findings, *T*. *divaricata* should be domesticated due to its rich nutritional value.

## Figures and Tables

**Table 1:** The effect of potassium and pruning levels on plant height, flower bud number, and the fresh and dry weight of the flower buds of hydroponically grown *T. divaricata*.

K_2_SO_4_	Pruning	Plant Height	Flower Bud No.	Fresh Flower Buds (g)	Dry Flower Buds (g)
0 M	Unpruned	72.14 ± 1.85 a	5.30 ± 0.63 ab	16.14 ± 2.34 bc	13.59 ± 2.16 b
	5 cm	31.22 ± 0.77 efg	2.80 ± 0.29 bcdef	8.01 ± 2.31 e	6.19 ± 2.11 d
	10 cm	30.29 ± 2.24 def	4.60 ± 0.69 bc	9.76 ± 2.65 e	8.30 ± 2.43 c
	15 cm	34.23 ± 1.40 cd	3.30 ± 0.58 bcdef	15.83 ± 3.23 c	13.71 ± 2.94 b
0.0072 M	Unpruned	78.91 ± 1.65 a	7.80 ± 0.49 a	22.95 ± 3.44 a	20.06 ± 3.14 a
	5 cm	37.00 ± 1.14 de	3.60 ± 0.37 bcde	5.83 ± 1.16 f	4.11 ± 094 de
	10 cm	44.27 ± 1.68 cd	4.70 ± 0.78 bc	17.75 ± 4.42 b	14.31 ± 3.83 b
	15 cm	49.43 ± 1.57 bc	7.60 ± 0.65 a	22.09 ± 4.01 a	19.54 ± 3.65 a
0.0144 M	Unpruned	55.57 ± 2.28 b	4.00 ± 0.52 bcd	11.06 ± 2.22 de	7.93 ± 1.85 c
	5 cm	27.71 ± 0.85 efg	1.80 ± 0.47 defg	5.54 ± 2.32 f	4.16 ± 1.86 de
	10 cm	25.50 ± 3.59 fgh	2.70 ± 0.67 cdefg	9.28 ± 1.92 e	7.35 ± 1.66 c
	15 cm	27.52 ± 4.74 efg	1.00 ± 0.45 fg	4.95 ± 2.05 f	3.64 ± 1.54 e
0.0216 M	Unpruned	49.19 ± 2.43 bc	2.70 ± 0.34 cdefg	8.75 ± 1.11 e	6.04 ± 0.85 d
	5 cm	13.11 ± 2.80 i	0.20 ± 0.13 g	0.42 ± 0.42 g	0.23 ± 0.23 f
	10 cm	15.29 ± 2.24 hi	1.30 ± 0.54 g	2.49 ± 1.28 g	1.45 ± 0.45 f
	15 cm	21.51 ± 0.63 hi	1.10 ± 0.28 g	1.64 ± 0.5 g	0.86 ± 0.30 f
		Two-way ANOVA F-Statistic			
K_2_SO_4_		17,272 *	483.67 *	4275 *	3495 *
Pruning		34,194 *	158.22 *	1903 *	1411 *
K_2_SO_4_ × Pruning		1582 *	95.61 *	1156 *	1018 *

Note: Means were ranked along the column using Tukey’s LSD at α level of 0.05. Means that do not share a letter are significantly different. *: significant at *p* ≤ 0.05.

**Table 2:** The effect of potassium and pruning levels on the fresh and dry weight of shoots and roots of hydroponically grown *T. divaricata*.

K_2_SO_4_	Pruning	Shoot Fresh Weight (g)	Shoot Dry Weight (g)	Root Fresh Weight (g)	Root Dry Weight (g)
0 M	Unpruned	93.48 ± 19.3 bc	16.41 ± 2.3 b	51.30 ± 2.39 bc	27.89 ± 1.31 abcd
	5 cm	60.4 ± 9.33 bcde	6.72 ± 1.76 d	30.98 ± 1.20 de	21.27 ± 1.72 def
	10 cm	69.8 ± 12.5 cd	17.27 ± 3.75 b	30.86 ± 1.13 de	22.80 ± 1.78 cde
	15 cm	114.5 ± 7.79 ab	19.19 ± 4.8 ab	46.06 ± 1.41 cd	34.08 ± 2.25 abc
0.0072 M	Unpruned	154.3 ± 12.6 a	19.38 ± 3.76 a	63.23 ± 2.68 a	38.17 ± 3.81 a
	5 cm	51.65 ± 4.96 d	8.65 ± 2.14 cd	30.98 ± 1.20 ef	25.60 ± 0.84 bcd
	10 cm	91.0 ± 13.8 bc	8.76 ± 2.51 cd	21.22 ± 1.26 e	34.86 ± 1.33 def
	15 cm	89.5 ± 16.0 bc	15.80 ± 3.22B	38.09 ± 2.31 de	26.84 ± 2.85 abcd
0.0144 M	Unpruned	97.9 ± 17.4 b	11.13 ± 2.25 bc	51.77 ± 3.05 bc	35.78 ± 3.76 ab
	5 cm	25.80 ± 5.56 f	6.79 ± 2.38 d	59.92 ± 3.30 ab	21.27 ± 1.72 bcd
	10 cm	45.0 ± 12.4 e	9.28 ± 1.92 cd	30.50 ± 3.59 ef	17.34 ± 2.60 def
	15 cm	50.0 ± 15.6 de	4.95 ± 2.05 e	22.93 ± 2.00 fg	12.17 ± 2.83 efg
0.0216 M	Unpruned	21.53 ± 4.10 f	8.75 ± 1.11 cd	32.88 ± 1.33 ef	10.91 ± 1.67 fg
	5 cm	5.91 ± 2.26 g	0.42 ± 0.42 e	14.32 ± 0.34 g	2.62 ± 0.89 g
	10 cm	3.81 ± 1.82 g	2.49 ± 1.28 e	19.19 ± 0.58 g	3.45 ± 1.57 g
	15 cm	4.47 ± 3.67 g	1.64 ± 0.57 e	24.49 ± 0.95 fg	1.95 ± 1.41 g
		Two-way ANOVA F-Statistic			
K_2_SO_4_		18,136 *	3541 *	11,307 *	13,755 *
Pruning		80,109 *	1276 *	8830 *	3393 *
K_2_SO_4_ × Pruning		23,891 *	1017 *	9949 *	2825 *

Note: Means were ranked along the column using Tukey’s LSD at α level of 0.05. Means that do not share a letter are significantly different. *: significant at *p* ≤ 0.05.

**Table 3:** (**a**,**b**) The effect of potassium dosage and pruning levels on macronutrients of flower buds of hydroponically grown *T. divaricata*.

**(a)**								
**K_2_SO_4_**	**Pruning**	**Ca** **(mg/100 g DW)**	**Mg** **(mg/100 g DW)**	**Na** **(mg/100 g DW)**	**P** **(mg/100 g DW)**	**N** **(mg/100 g DW)**	**K** **(mg/100 g DW)**	**K/Ca + Mg (mg/100 g DW)**
0 M	Unpruned	610 ± 0.01 a	340 ± 0.01 ab	220 ± 0.00 c	740 ± 0.01 ab	3790 ± 0.07 b	5700 ± 0.01 hi	2530 ± 0.05 h
	5 cm	480 ± 0.01 a	340 ± 0.01 ab	190 ± 0.00 cde	700 ± 0.01 abc	3630 ± 0.07 b	5460 ± 0.02 ij	2580 ± 0.09 h
	10 cm	650 ± 0.01 a	370 ± 0.02 a	180 ± 0.01 def	700 ± 0.01 abc	3800 ± 0.00 b	5130 ± 0.01 jk	2030 ± 0.017 i
	15 cm	640 ± 0.01 a	350 ± 0.00 a	210 ± 0.01 cd	660 ± 0.02 cde	3690 ± 0.05 b	5130 ± 0.09 jk	2160 ± 0.00 i
0.0072 M	Unpruned	260 ± 0.01 a	170 ± 0.01 e	120 ± 0.01 h	670 ± 0.01 cd	3550 ± 0.08 b	9460 ± 0.78 a	9490 ± 0.02 a
	5 cm	440 ± 0.01 a	280 ± 0.28 bc	130 ± 0.00 gh	750 ± 0.01 a	3690 ± 0.05 b	6490 ± 0.03 ef	3750 ± 0.05 g
	10 cm	250 ± 0.01 a	210 ± 0.01 cde	120 ± 0.00 gh	710 ± 0.01 abc	3770 ± 0.06 b	7090 ± 0.04 cd	5680 ± 0.14 b
	15 cm	390 ± 0.01 a	250 ± 0.01 cd	130 ± 0.01 gh	740 ± 0.01 ab	3700 ± 0.058 b	7530 ± 0.07 b	7530 ± 0.07 d
0.0144 M	Unpruned	280 ± 0.01 a	240 ± 0.01 cde	270 ± 0.01 b	670 ± 0.24 cd	4300 ± 0.06 a	6460 ± 0.03 ef	4930 ± 0.04 d
	5 cm	330 ± 0.01 a	250 ± 0.01 cd	140 ± 0.01 fgh	660 ± 0.15 cde	4340 ± 0.02 a	5820 ± 0.08 h	4180 ± 0.04 ef
	10 cm	310 ± 0.01 a	260 ± 0.00 cd	340 ± 0.01 a	760 ± 0.01 a	4470 ± 0.09 a	7160 ± 0.07 bc	4950 ± 0.01 d
	15 cm	270 ± 0.01 a	230 ± 0.00 cde	180 ± 0.01 cde	620 ± 0.01 def	4300 ± 0.06 b	6730 ± 0.03 def	5350 ± 0.02 c
0.0216 M	Unpruned	230 ± 0.01 a	220 ± 0.01 cde	270 ± 0.01 b	590 ± 0.01 ef	2970 ± 0.03 cd	6360 ± 0.04 fg	3910 ± 0.06 fg
	5 cm	700 ± 10.2 a	210 ± 0.01 cde	130 ± 0.01 gh	680 ± 0.02 bcd	3530 ± 0.145 b	3530 ± 0.15 k	4150 ± 0.02 ef
	10 cm	500 ± 0.88 a	270 ± 0.03 bc	300 ± 0.01 b	560 ± 0.02 cde	3170 ± 0.07 c	6770 ± 0.13 de	4400 ± 0.05 e
	15 cm	240 ± 0.01 a	190 ± 0.01 de	160 ± 0.01 efg	550 ± 0.02 f	2800 ± 0.06 d	6070 ± 0.027 fg	4760 ± 0.04 d
Two-way ANOVA F-Statistic								
K_2_SO_4_		1.06 ns	75.85 *	137.70 *	43.30 *	181.01 *	706.71 *	2898 *
Pruning		0.96 ns	5.40 *	100.60 *	19.48 *	21.50 *	228.54 *	519 *
K_2_SO_4_ × pruning		0.95 ns	4.72 *	45.47 *	11.32 *	9.99 *	92.39 *	532 *
(**b**)								
**K_2_SO_4_**	**Pruning**	**Zn** **(mg/100 g DW)**		**Mn** **(mg/100 g DW)**		**Cu** **(mg/100 g DW)**	**Fe** **(mg/100 g DW)**	
0 M	Unpruned	10.8 ± 1.15 bcd		4.23 ± 1.20 def		0.5. ± 0.00 a	12.3 ± 0.88 abc	
	5 cm	8.9 ± 0.58 ef		2.9 ± 0.88 jk		0.373 ± 0.15 bc	12.23 ± 0.67 bc	
	10 cm	8.4. ± 1.15 fg		3.36 ± 0.33 ij		0.464 ± 0.31 ab	8.9 ± 0.58 fg	
	15 cm	10.2 ± 1.15 bcd		3.8 ± 1.00 gh		0.5 ± 0.00 a	8.72 ± 0.64 fg	
0.0072 M	Unpruned	10.9 ± 1.53 bc		6.7 ± 0.58 a		0.2 ± 0.00 d	9.23 ± 2.03 ef	
	5 cm	8.6 ± 0.88 f		4.4 ± 1.15 de		0 ± 0.00 f	7.1 ± 0.58 h	
	10 cm	7.3 ± 0.58 h		4.9 ± 0.58 bc		0.1 ± 0.00 e	5.6 ± 1.53 i	
	15 cm	11 ± 0.58 b		5.2 ± 1.20 b		0.1 ± 0.00 e	8.2 ± 1.13 g	
0.0144 M	Unpruned	10.8 ± 1.53 bcd		3.8 ± 0.58 gh		0.4 ± 0.00 bc	13 ± 0.58 ab	
	5 cm	10.1 ± 0.58 bcd		4.2 ± 0.58 efg		0.4 ± 0.00 bc	8.1 ± 1.15 g	
	10 cm	12.6 ± 0.88 a		4.03 ± 0.35 efgh		0.4 ± 0.00 bc	12 ± 4.51 c	
	15 cm	9.8 ± 0.58 de		2.9 ± 0.58 k		0.2 ± 0.00 d	13.3 ± 2.65 a	
0.0216 M	Unpruned	10.16 ± 1.67 bcd		3.9 ± 0.58 fgh		0.537 ± 0.34 a	10.7 ± 3.71 d	
	5 cm	9.83 ± 6.94 cde		4.633 ± 0.88 cd		0.357 ± 0.32 c	8.8 ± 0.56 fg	
	10 cm	8.86 ± 0.33 ef		3.7 ± 0.58 hi		0.370 ± 0. 21 bc	9.43 ± 0.89 ef	
	15 cm	7.5 ± 2.52 gh		2.8 ± 0.00 k		0.467 ± 0.41 ab	10.13 ± 0.89 de	
Two-way ANOVA F-Statistic								
K_2_SO_4_		2017.8 *		2364.29 *		96.497 *	10,710.7 *	
Pruning		1456.4 *		597. 59 *		10.313 *	4358.4 *	
K_2_SO_4_ × pruning		5253.8 *		1365.23 *		14.407 *	7284.4 *	

Note: Means were ranked along the column using Tukey’s LSD at α level of 0.05. Means that do not share a letter are significantly different. ns: no significance. *: significant at *p* ≤ 0.05.

**Table 4:** The effect of potassium and pruning levels on proximate composition of flower buds of hydroponically grown *T. divaricata*.

K_2_SO_4_	Pruning Levels	% Ash	% Crude Fat	% Crude Protein	% NDF	% NFC	% Moisture	Energy Value (KJ/100 g)
0 M	Unpruned	15.7 ± 0.02 bcd	2.2 ± 0.05 abc	24.2 ± 0.04 cd	33.2 ± 0.2 cd	24.6 ± 0.2 def	10.1 ± 0.04 g	1476.4 ± 0.3 ab
	5 cm	14.9 ± 0.20 cd	2.3 ± 0.08 abc	23.6 ± 0.04 cde	31.8 ± 0.4 ef	27.2 ± 0.4 bcd	10.3 ± 0.1 efg	1491.1 ± 2.2 ab
	10 cm	14.5 ± 0.20 d	2.0 ± 0.05 bc	24.1 ± 0.01 cd	34.6 ± 0.1 bc	24.7 ± 01 de	10.5 ± 0.07 defg	1493.5 ± 6 a
	15 cm	14.0 ± 2.30 d	2.0 ± 0.02 bc	23.46 ± 0.1 de	35.3 ± 0.2 b	25.1 ± 2.5 de	10.23 ± 0.08 fg	1503.2 ± 3.2 a
0.0072 M	Unpruned	23.6 ± 0.30 a	1.9 ± 0.02 bc	22.3 ± 0.2 ef	37.8 ± 0.2 a	14.2 ± 0.4 g	10.9 ± 0.03 cde	1335.7 ± 0.5 d
	5 cm	16.0 ± 2.10 bcd	2.0 ± 0.01 bc	23.7 ± 0.4 cde	32.3 ± 0.3 de	25.8 ± 2.1 cde	10.8 ± 0.1 cdef	1469 ± 0.3 abc
	10 cm	19.1 ± 0.05 bc	2.1 ± 0.06 abc	23.7 ± 0.1 cde	31.6 ± 0.3 ef	23.3 ± 0.2 def	12.2 ± 0.1 a	1417.4 ± 0.2 bc
	15 cm	20.0 ± 0.04 ab	1.8 ± 0.01 bc	23.19 ± 0.2 def	35.05 ± 0.08 b	19.9 ± 0.3 f	11.8 ± 0.08 ab	1395 ± 0.4 cd
0.0144 M	Unpruned	16.5 ± 0.30 bcd	2.3 ± 0.08 ab	25.1 ± 0.3 bc	31.94 ± 0.08 def	24.0 ± 0.6 def	11.4 ± 0.2 bc	1464.9 ± 4 abc
	5 cm	14.8 ± 0.10 cd	1.9 ± 0.02 bc	26.6 ± 0.3 a	30. ± 0.1 gh	26.5 ± 0.2 cde	11.8 ± 0.01 ab	1486.8 ± 1.8 ab
	10 cm	16.8 ± 0.01 bcd	2.3 ± 0.1 ab	26.0 ± 0.1 ab	32.6 ± 0.3 de	22.1 ± 0.4 ef	11.5 ± 0.1 bc	1459.9 ± 2.1 abc
	15 cm	17.1 ± 0.07 bcd	2.6 ± 0.29 a	23.5 ± 0.3 de	31 ± 0.01 fg	25.7 ± 0.02 cde	11.0 ± 0.06 cd	1461.2 ± 2.1 abc
0.0216 M	Unpruned	15.7 ± 0.20 bcd	2.1 ± 0.08 abc	22.5 ± 0.3 ef	26.9 ± 0.1 j	59.5 ± 0.5 abc	10.9 ± 0.03 cde	1475.5 ± 2.1 ab
	5 cm	13.3 ± 0.10 d	1.7 ± 0.06 c	24.3 ± 0.2 cd	30.1 ± 0.04 gh	60.5 ± 0.2 a	10.2 ± 0.1 fg	1507.3 ± 3.1 a
	10 cm	15.6 ± 0.20 cd	1.9 ± 0.01 bc	23.2 ± 0.2 de	28.9 ± 0.2 hi	59.0 ± 0.1 abc	9.9 ± 0.2 g	1473.2 ± 3.6 ab
	15 cm	16.2 ± 0.30 bcd	1.9 ± 0.1 bc	21.7 ± 0.5 f	28.2 ± 0.3 ij	60.1 ± 0.4 ab	10.1 ± 0.1 fg	1462.2 ± 3.3 abc
Two-way ANOVA F-Statistic								
K_2_SO_4_		28.05 *	10.56 *	56.51 *	394 *	85.82 *	103.53 *	29.46 *
Pruning		9.59 *	1.32 ns	26.97 *	24.2 *	10.59 *	3.04 *	8.56 *
K_2_SO_4_ × pruning		3.02 *	3.80 *	6.31 *	58.38 *	8.85 *	14.15 *	3.18 *

* NDF = Neutral Detergent fibre; NFC = Non-Fibre Carbohydrates, ns = not significant. Note: Means were ranked along the column using Tukey’s LSD at α level of 0.05. Means that do not share a letter are significantly different. *: significant at *p* ≤ 0.05.

**Table 5:** The effect of potassium concentration and pruning level on phytochemicals and the antioxidant activity of flower buds of *T. divaricata*.

K_2_SO_4_	Pruning Levels	Polyphenols (mg GAE/g)	Flavonols (mg QE/g)	FRAP (µmol AAE/g)	DPPH (µmol TE/g)	TEAC (µmol TE/g)
0 M	Unpruned	3.55 ± 0.15 de	3.59 ± 0.09 def	14.98 ± 0. 14 gh	5.04 ± 0.02 fg	4.45 ± 0.09 gh
	5 cm	3.04 ± 0.07 ef	0.69 ± 0.03 j	15.33 ± 0.48 gh	5.37 ± 0.18 efg	2.69 ± 0.06 j
	10 cm	4.63 ± 0.14 bc	1.83 ± 0.12 hi	23.02 ± 0.80 cd	7.36 ± 0.23 cd	4.32 ± 0.19 gh
	15 cm	4.29 ± 0.25 bcd	1.99 ± 0.08 hi	20.09 ± 0.86 de	8.63 ± 0.16 bc	19.04 ± 0.49 a
0.0072 M	Unpruned	6.55 ± 0.05 a	3.33 ± 0.08 ef	19.27 ± 0.71 ef	6.44 ± 0.18 de	5.52 ± 0.06 def
	5 cm	5.84 ± 0.08 a	3.88 ± 0.21 cde	33.69 ± 1.06 a	9.34 ± 0.18 bc	5.12 ± 0.03 efgh
	10 cm	6.13 ± 0.29 a	5.22 ± 0.27 a	29.48 ± 0.85 b	11.47 ± 0.42 a	5.87 ± 0.31 b
	15 cm	6.33 ± 0.15 a	4.56 ± 0.29 abc	28.34 ± 0.63 b	9.25 ± 0.36 b	5.87 ± 0.31 bc
0.0144 M	Unpruned	6.57 ± 0.19 a	2.14 ± 0.04 hi	10.09 ± 0.55 i	3.54 ± 0.09 h	5.23 ± 0.16 efg
	5	2.69 ± 0.13 f	4.29 ± 0.21 cd	16.31 ± 0.38 fg	6.37 ± 0.21 de	6.74 ± 0.07 c
	10	4.26 ± 0.07 cd	4.33 ± 0.08 bc	24.14 ± 0.92 c	9.01 ± 0.32 b	6.35 ± 0.27 cd
	15	5.02 ± 0.13 b	5.05 ± 0.09 ab	19.46 ± 0.23 ef	6.18 ± 0.22 def	4.17 ± 0.07 hi
0.0216 M	Unpruned	4.13 ± 0.15 cd	2.47 ± 0.09 gh	16.52 ± 0.71 fg	6.39 ± 0.17 de	3.27 ± 0.14 ij
	5 cm	2.93 ± 0.06 ef	3.04 ± 0.05 fg	10.99 ± 0.29 i	5.59 ± 0.47 ef	6.82 ± 0.09 c
	10 cm	3.15 ± 0.02 ef	1.53 ± 0.08 hi	12.48 ± 0.55 hi	4.31 ± 0.07 gh	4.57 ± 0.06 fgh
	15 cm	5.23 ± 0.16 ef	1.82 ± 0.06 hi	12.52 ± 0.44 hi	4.10 ± 0.09 gh	3.29 ± 0.08 ij
Two-way ANOVA F-Statistics						
K_2_SO_4_		36.97 *	47.58 *	726.08 *	68.19 *	74.58 *
Pruning		26.77 *	1.20 *	704.83 *	57.81 *	187.34 *
K_2_SO_4_ × Pruning		26.56 *	36.38 *	751.74 *	97.04 *	476.14 *

Note: Means were ranked along the column using Tukey’s LSD at α level of 0.05. Means that do not share a letter are significantly different. *: significant at *p* ≤ 0.05.

**Table 6:** Arrangement of each gutter on four NFT systems with different pruning levels (cm) and potassium sulphate (mL) fertigation.

NFT/Table	Gutter 1	Gutter 2	Gutter 3	Gutter 4
1	5 cm + 0 M	10 cm + 0 M	15 cm + 0 M	Unpruned + 0 M
2	5 cm + 0.0072 M	10 cm + 0.0072 M	15 cm + 0.0072 M	Unpruned + 0.0072 M
3	5 cm + 0.0144 M	10 cm + 0.0144 M	15 cm + 0.0144 M	Unpruned + 0.0144 M
4	5 cm + 0.0216 M	10 cm + 0.0216 M	15 cm + 0.0216 M	Unpruned + 0.0216 M

NFT = Nutrient Filter Technique hydroponic system.

## Data Availability

All data is included in the manuscript.
